# Developmental remodelling of *Drosophila* flight muscle sarcomeres: a scaled myofilament lattice model based on multiscale morphometrics

**DOI:** 10.1098/rsob.250182

**Published:** 2025-08-13

**Authors:** Péter Görög, Tibor Novák, Tamás F. Polgár, Péter Bíró, Adél Gutheil, Csaba Kozma, Tamás Gajdos, Krisztina Tóth, Alexandra Tóth, Miklós Erdélyi, József Mihály, Szilárd Szikora

**Affiliations:** ^1^ HUN-REN Biological Research Centre, Szeged, Hungary; ^2^ Department of Optics and Quantum Electronics, University of Szeged, Szeged, Hungary; ^3^ Transmission Electron Microscope Laboratory, Core Facility, Szeged, Hungary; ^4^ Doctoral School of Theoretical Medicine, University of Szeged, Szeged, Hungary; ^5^ Department of Genetics, University of Szeged, Szeged, Hungary

**Keywords:** flight muscle, sarcomere, *Drosophila*, thin filament, thick filament, nanoscopy

## Abstract

The indirect flight muscle is a widely used model for studying sarcomere structure and muscle development due to its extremely regular architecture. Nevertheless, precise measurement of the basic sarcomeric parameters remains a challenge even in this greatly ordered tissue. In this study, we identified several factors affecting measurement reliability and developed a software tool for precise, high-throughput measurement of sarcomere length and myofibril width. The accuracy of this new tool was validated against simulated images and blinded manual measurements. To extend the scope of this morphometric analysis to the sub-sarcomeric scale, we used electron and super-resolution microscopy to quantify myofilament number and filament length during myofibrillogenesis. Our findings revealed the dynamics of thin and thick filament elongation, as well as the addition of myofilaments at the sarcomere periphery during myofibrillogenesis. We precisely measured the dimensions of the Z-disc, I-band and H-zone during development, enabling us to construct refined models of sarcomere growth at the level of individual myofilaments, providing a spatial framework for interpreting nanoscopic localization data. These models deepen our understanding of sarcomere growth and lay the groundwork for future studies on the molecular mechanisms driving myofilament elongation and assembly.

**Figure:**
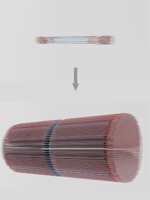

## 1. Introduction

The indirect flight muscles (IFMs) of *Drosophila* provide an excellent model for studying muscle organization and the molecular processes of myogenesis. Over the years, significant research, combined with advances in imaging techniques and the wide range of functional genomic tools available in *Drosophila* [1–6], has made the IFM one of the most well-studied muscle systems. Techniques like fluorescent nanoscopy can now reveal the detailed composition and arrangement of densely packed sarcomeric regions. Single-molecule localization microscopy, along with structural averaging, can pinpoint sarcomeric protein positions with precision under 10 nm, and has been used to reconstruct large sarcomeric protein complexes [7–10]. Additionally, *in situ* cryo-electron tomography and cryo-focused ion beam scanning electron microscopy have been applied in other model systems [11,12] and show strong potential for enabling high-resolution molecular and structural analysis of IFM myofilaments in their native state in the near future.

The combination of highly regular structure, uniformity and synchronized growth during pupal development with the tools available in *Drosophila* makes the IFM an ideal system for dissecting the molecular mechanisms of thin- and thick-filament elongation and assembly during myofibrillogenesis. While some of the molecular components involved in this process have been identified [10,13–17], a comprehensive structural model of myofilament growth is still missing. Such a model must integrate three core parameters across the entire developmental timeline: (i) the length of the myofilaments; (ii) their quantity; and (iii) their spatial arrangement relative to each other and to the sarcomere’s structural landmarks. Existing studies offer some direct measurements, but these are rarely comprehensive [13,16,18–21]. Additionally, while some parameters could be inferred from existing data, we found that published values are often contradictory, which limits their usefulness in constructing accurate, scaled models of myofilament organization.

In this study, we provide a detailed analysis of IFM sarcomere growth. Building on previous work, we summarize the current knowledge on myofilament lattice morphometrics of IFM sarcomeres while addressing discrepancies in the literature. We also highlight the importance of measurement conditions and the need for precise reporting. Furthermore, we introduce a software tool that automates reliable size measurements from micrographs of IFM myofibrils. Finally, using a combination of conventional fluorescence microscopy, single-molecule localization microscopy and transmission electron microscopy (TEM), we present scaled models of the myofilament lattice of IFM sarcomeres, detailing the average number and length of the myofilaments, their relative position, as well as the dimensions of the sarcomeric bands and zones during myofibrillogenesis.

## 2. Results and discussion

### 2.1. Discrepancies in reported IFM sarcomere morphometrics

**Figure 1.**
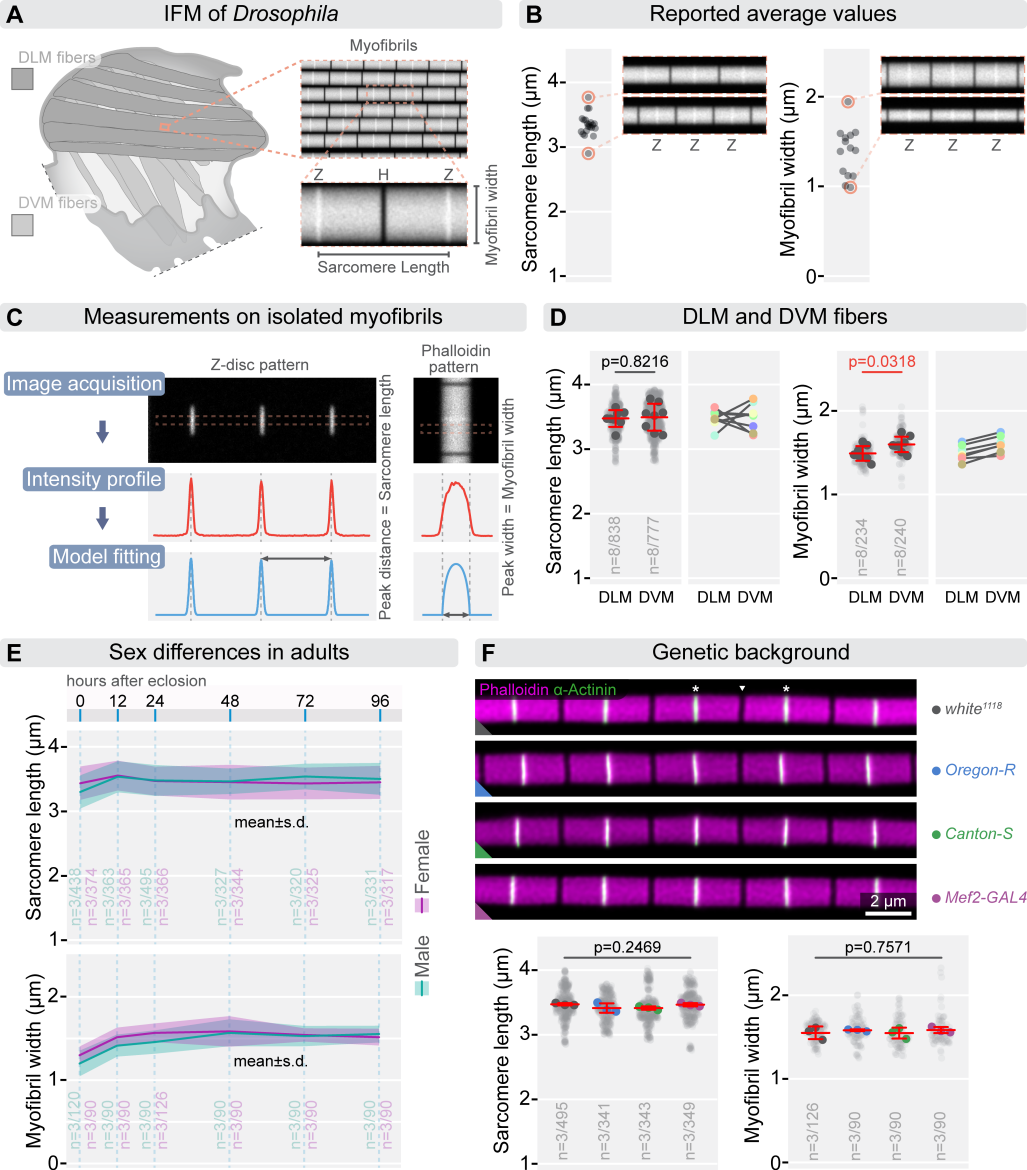
Morphometric measurements of IFM sarcomeres. (A) Schematic illustrating that indirect flight muscles (IFMs) are made up of the dorsal longitudinal muscles (DLM, shown in darker grey) and the Dorso-Ventral muscles (DVM, shown in lighter grey). The myofibrils consist of regular, uniform sarcomeres with characteristic lengths and diameters. The Z-discs (Z) at the borders of sarcomeres and the central H-zone (H) are labelled. (B) Plots displaying previously reported averages for sarcomere length and diameter, along with simulated images on the right that highlight the differences of the published measurements. (C) Panels outlining the process for measuring sarcomere length and diameter. Sarcomere length is determined from images where the Z-disc is labelled, while myofibril diameter is measured from phalloidin-labelled samples. After image acquisition, an intensity profile is recorded either along or perpendicular to the myofibril. The red dashed lines border the ROI used for generating the intensity profiles. This profile is then fitted with the appropriate Gaussian or Disc Model function to calculate sarcomere length and myofibril diameter, respectively. (D) A comparison of sarcomere length and diameter between DLM and DVM fibres reveals that while sarcomere lengths in these fibres are not significantly different (*p* = 0.8216), DVM fibres consistently exhibit larger diameters in every tested animal (*p* = 0.0318). Panels on the right show connected mean values for DLM and DVM fibres from the same animal. Statistical analysis was performed using an unpaired *t*‐test. (E) Panels display the measured differences in sarcomere length and myofibril width between sexes after eclosion. Sarcomere length and myofibril width were compared between males and females from 0 to 96 h AE using a two-way ANOVA with Sidak’s multiple comparisons test. Sarcomere length showed no significant differences between sexes (*p* = 0.9846) or across developmental time points (*p* = 0.1533). By contrast, myofibril width varied significantly between sexes (*p* = 0.0374) and over post-eclosion development (*p* < 0.0001). (F) The micrographs display representative images of isolated myofibrils from flies with different genetic backgrounds, labelled with phalloidin (magenta) and α-actinin (green). The accompanying plots indicate no significant differences in the IFM morphometrics of these myofibrils. Statistical analysis was conducted using one-way ANOVA with Tukey’s multiple comparison test. In panels (D) and (F) light grey dots represent individual measurements of sarcomere length and myofibril diameter, while the larger dots indicate the mean values from independent experiments. Error bars represent the mean and s.d. of independent experiments. ‘*n*’ refers to the number of independent experiments/the number of individual measurements. In panel (F), white asterisks indicate the Z-discs of an exemplary sarcomere, and the position of the M-line is shown by a white triangle. Raw data used to generate the plots presented in this figure are available in the source data file (electronic supplementary material, Fig1SourceData).

Flight in *Drosophila* is driven by the coordinated action of the two main muscle groups of the IFM, the dorsal longitudinal muscles (DLMs) and the dorso-ventral muscles (DVMs) (figure 1A). The contractile machinery of the fibrillar IFM consists of uniform, cylindrical myofibrils extending along the entire length of the muscle fibres. Each myofibril is composed of hundreds of serially connected, nearly uniform-sized sarcomeres (figure 1A) [22,23]. Consequently, sarcomere length and myofibril width (or diameter) are frequently reported morphometric traits.

Despite the remarkable regularity of IFM sarcomeres, various laboratories tend to report significantly different values, with average sarcomere lengths ranging from 2.9 µm to 3.77 µm and myofibril widths from 0.98 µm to 1.94 µm in adult flies (figure 1B) [1,8,13,16,17,20,21,23–38]. To address these discrepancies, we conducted a review of the published measurement methods. This analysis revealed several significant factors contributing to the variability in reported measurements.

### 2.2. An automated method to accurately measure the sarcomeric parameters using isolated individual myofibrils

First, we decided to assess the accuracy and precision of our routinely used measurement methods. Sarcomeres are bordered by Z-discs, and by definition, their length is equal to the distance between two adjacent Z-lines (the midlines of the Z-discs). Z-disc markers, such as α-actinin or ZASPs (Z band alternately spliced PDZ-containing protein), are typically used to label these discs, enabling relatively simple measurements from confocal micrographs (figure 1C). The other key parameter—myofibril diameter—is typically measured using phalloidin staining. However, accurately delineating their boundaries in micrographs is difficult—even under optimal conditions (high signal-to-noise ratio, no overlapping fibres, etc.; figure 1C). This limitation arises from the fundamental nature of light microscopy as the image produced is a blurred version of the actual structure, due to convolution with the microscope’s point spread function.

For our analysis, we opted to use isolated individual myofibrils rather than whole-mount sectioned or microdissected muscle fibres (used as controls) because they offer clear advantages for accurate sarcomere length and diameter measurements. (i) Simplicity and reproducibility: IFM myofibrils can be isolated and mounted with minimal training and without specialized sectioning tools, yielding highly consistent results. By contrast, microdissection demands more experience, while whole-mount sectioning demands both experience and expensive equipment, which can limit throughput and introduce variability. (ii) Optimal imaging geometry: once isolated, myofibrils lie flat on the coverslip, aligning with the focal plane of the objective lens. This orientation allows for high-resolution, undistorted imaging and accurate two-dimensional measurements, free from interference by neighbouring biological structures (e.g. other myofibrils). In other preparations, myofibrils often overlap or lie in close proximity, making it difficult to resolve individual myofibrils and reliably measure their width. In addition, sectioned surfaces can be damaged during cutting, necessitating deeper imaging that may introduce optical artifacts due to refractive index mismatches. (iii) High-throughput, unbiased sampling: images of isolated myofibrils are straightforward to analyse automatically. These preparations also yield randomly sampled myofibrils, potentially from multiple animals, enhancing the statistical robustness of the measurements. (iv) Validated accuracy (detailed in the following §2): we demonstrate that sarcomere lengths measured from isolated myofibrils match very well to those obtained from whole-mount sectioned or microdissected preparations in both mature and early pupal stages, directly refuting any concern that mechanical isolation might distort sarcomere length measurements. Although diameter measurements from isolated myofibrils are somewhat more sensitive to sample handling, the contributing factors are characterized and have been addressed in previous work, and importantly, we show here that our preparation method yields results that align well with the theoretical *in vivo* myofibril diameter.

To determine the precision and accuracy of sarcomere length and myofibril diameter measurements, we generated artificial images of IFM myofibrils with known dimensions, simulating the image formation process (electronic supplementary material, figure S1A). Since most previously reported data were obtained through manual measurements, we conducted a blinded test to evaluate their reliability. This test revealed significant variability between individuals, often considerably underestimating myofibril diameter (electronic supplementary material, figure S1D).

**Figure 2.**
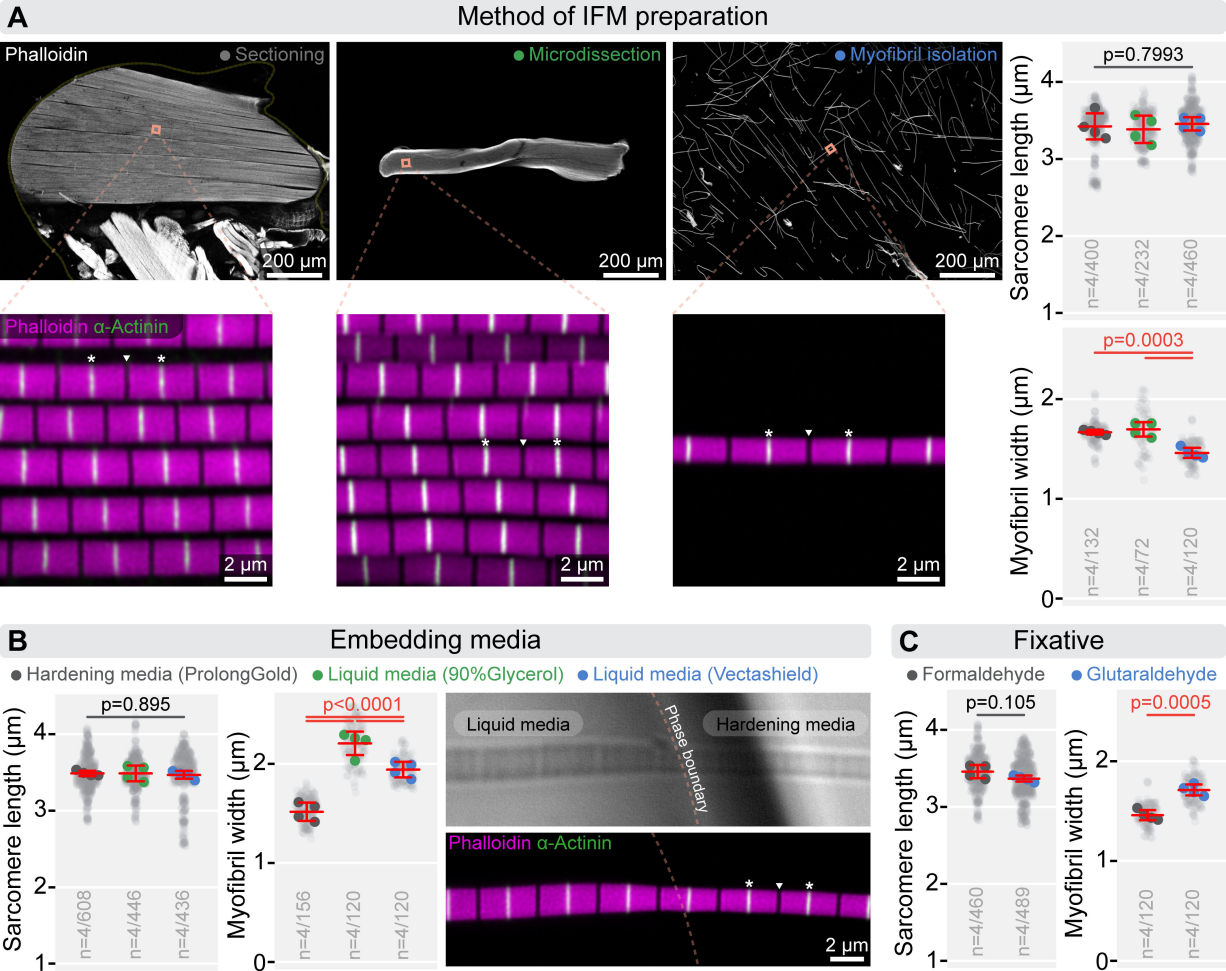
Impact of sample preparation methods on IFM morphometric analysis. (A) Low- and high-resolution images illustrate commonly used methods for preparing IFM samples. F-actin is stained with phalloidin (grey or magenta), and Z-discs are labelled with α-actinin (green). The impact of these preparation methods on sarcomere morphometrics is compared. While sarcomere lengths do not differ significantly between methods (*p* = 0.799), the diameter of individual myofibrils is significantly smaller in isolated myofibrils compared with sectioned or microdissected samples (*p* = 0.0003). Statistical analysis was conducted using one-way ANOVA with Tukey’s multiple comparison test. (B) Panels highlight how embedding media affect sarcomere length and myofibril diameter. While sarcomere length remains unchanged (*p* = 0.895), myofibril diameter is highly sensitive to the medium used, showing significant differences (*p* < 0.0001). Hardening media, such as ProLong Gold, reduce myofibril diameter, whereas liquid media, like glycerol-based solutions or Vectashield increase it. Micrographs (on the right) illustrate this effect: in the transmitted light image (top), a liquid media (90% Glycerol in PBS) bubble is visible within the hardening medium (ProLong Gold), and in the fluorescence image (bottom), variations in myofibril diameter are apparent. F-actin is stained with phalloidin (magenta), while Z-discs are labelled with α-actinin (green). Statistical analysis was performed using one-way ANOVA with Tukey’s multiple comparison test. (C) Panels show the effects of formaldehyde and glutaraldehyde on sarcomere length and myofibril diameter. While fixative choice does not significantly impact sarcomere length (*p* = 0.105), myofibrils fixed with glutaraldehyde are significantly thicker than those fixed with formaldehyde (*p* = 0.0005). An unpaired *t*‐test was used for analysis. In panels (A), (B) and (C) light grey dots represent individual measurements of sarcomere length and myofibril diameter, while the larger dots indicate the mean values from independent experiments. Error bars represent the mean and s.d. of independent experiments. ‘*n*’ refers to the number of independent experiments/number of individual measurements. In panels (A) and (B), white asterisks indicate the Z-discs of an exemplary sarcomere, and the position of the M-line is shown by a white triangle. Raw data used to generate the plots presented in this figure are available in the source data file (electronic supplementary material, Fig2SourceData).

To perform these measurements efficiently and with high accuracy, we developed a dedicated, user-friendly software tool called IMA (Individual Myofibril Analyser). IMA automatically segments myofibrils from Z-stacks of raw images containing both phalloidin and Z-disc labelling. It uses spline fitting and applies appropriate model functions to intensity profiles to accurately and precisely calculate both sarcomere length and myofibril width (figure 1C; electronic supplementary material, figure S2). For sarcomere length, IMA analyses intensity profiles along the myofibril in the Z-disc channel, fitting multiple Gaussian functions to determine peak-to-peak distances, which correspond to sarcomere length. To estimate myofibril diameter, it extracts intensity profiles perpendicular to the myofibril in the phalloidin channel and fits a disc function to generate precise width measurements (figure 1C; electronic supplementary material, figure S2) (further details are provided in the IMA User Guide and §5). The accuracy and precision of the tool were validated using our simulated IFM myofibril dataset (electronic supplementary material, figure S1D). In practical applications, this software not only improves measurement accuracy and precision but also significantly reduces analysis time by 15 to 20 folds, hence subsequently, we applied this new tool for our individual myofibril measurements. Importantly, we also note that although IMA was developed with the goal to optimize it for isolated myofibrils, in semi-automatic and manual mode it works for whole muscle preparations as well (figure 2A), with some reduction in processing efficiency.

Although several automated tools for sarcomere morphometric analysis have been introduced in recent years [1,39–45], none achieves the same level of accuracy and/or efficiency in measuring both sarcomere length and myofibril width on our dataset as IMA (electronic supplementary material, table S1). Moreover, many existing tools require programming expertise or manual input for image selection, which can be a significant bottleneck in the analysis workflow (electronic supplementary material, table S1).

### 2.3. IFM sarcomere morphometrics is affected by sex, age, fibre type and sample preparation

While reconsidering the literature, we noticed that the sex of the flies is often not specified, even though female pupae develop slightly faster than males, leading to earlier eclosion [46]. When we compared newly eclosed males and females, we found that males had slightly shorter sarcomeres, although this difference was not statistically significant (*p* = 0.9846), even if the trend was reproducible. This difference disappeared by 12 h AE, and sarcomere lengths remained similar thereafter (*p* = 0.1533; figure 1E). Likewise, myofibril width was significantly narrower in newly eclosed males (*p* = 0.0374), but this difference resolved between 24 and 48 h AE as the myofibrils grew in diameter (*p* < 0.0001; figure 1E). The IFMs consist of two muscle groups, the DLMs, which develop using larval muscle templates, and the DVMs, which form *de novo* [22,47]. Despite their different developmental origin, the sarcomeres of these muscles are generally considered structurally identical. To test this, we microdissected DLM and DVM fibres from adult females (24 h AE) and isolated individual myofibrils from them to compare their sizes. Our results showed that while sarcomere lengths were the same in both muscle groups, DVM myofibrils were consistently wider than those of the DLM (figure 1D). Different studies often utilize flies with different genetic background. To determine whether these genetic variations influence IFM morphometric measurements, we tested several commonly used strains (*white^1118^, Oregon-R* and *Canton-S* often used as wild type controls*,* and *Mef2-GAL4/+* as a frequently used muscle-specific Gal4 driver line), and found no significant differences in their sarcomeric parameters (figure 1F), indicating that the genetic background at this level has a negligible influence, and any of these strains can serve as an accurate control. Despite these findings, we trust that application of the best possible genetic matches will remain the most appropriate controls in future studies. Additionally, since the developmental rate of *Drosophila* is highly influenced by temperature, it is important to conduct experiments at a consistent temperature and ensure that comparisons are made under similar conditions.

Sample preparation introduces several variables that can potentially also influence IFM sarcomere morphometrics. Common preparation methods include whole-mount sectioning (via vibratome or cryosectioning), microdissection or the isolation of individual myofibrils. The tissue is typically fixed using an aldehyde-based fixative, such as formaldehyde or glutaraldehyde. After immunostaining, the samples are embedded in either a liquid or hardening medium.

We found that sarcomere length remains consistent regardless of the preparation method—whether whole-mount sectioning, microdissection or isolation of individual myofibrils (figure 2A)—as well as across different embedding approaches (figure 2B) (hardening or liquid media) and fixation conditions (figure 2C and electronic supplementary material, figure S1C) (duration and fixative type). By contrast, myofibril diameter is highly sensitive to these steps. Isolated myofibrils are significantly thinner than those in whole-mount sections or microdissected fibres (figure 2A). Expanding on the findings by DeAguero *et al*. [24], who reported that extended pre-fixation (approx. 24 h) alters morphometrics in cryosectioned IFM compared with brief post-fixation, we analysed isolated myofibrils fixed for 10, 20 (standard) or 60 min. While no differences were observed between the 10- and 20 min fixations, 60 min fixation led to a clear increase in myofibril diameter (electronic supplementary material, figure S1C), an effect that was also observed using a glutaraldehyde-based fixative (figure 2C). Finally, the choice of embedding medium profoundly affects myofibril thickness: using liquid media (90% glycerol/10% PBS or Vectashield) yields significantly thicker myofibrils than those embedded in hardening media such as ProLong Gold, while sarcomere length remained unaffected (figure 2B).

Why is IFM myofibril diameter so sensitive to sample preparation? Myofibril diameter is determined by two key factors: (i) the number of myofilaments, and (ii) the spacing between filaments (electronic supplementary material, figure S1B). While the number of myofilaments remains constant during sample processing, the inter-filament spacing is highly susceptible to changing conditions. For example, removal of the sarcolemma disrupts osmotic balance and allows excessive water influx into the lattice. This results in lattice overhydration, increased spacing between filaments and consequently, an expansion of myofibril diameter (electronic supplementary material, figure S1B [36,38,48]). Conversely, dehydration (required during EM sample preparation) cause the lattice to contract, significantly reducing filament spacing and leading to smaller myofibril diameters (electronic supplementary material, figure S1B; [21]). Additionally, hardening mounting media like ProLong Gold introduce further artefacts: during polymerization, these media shrink and exert compressive forces on the tissue [49], reducing lattice spacing and decreasing myofibril diameter (electronic supplementary material, figure S1B).

To determine the optimal protocol for accurate IFM myofibril morphometrics, we compared our measurements with *in vivo* filament arrangements inferred from small-angle X-ray scattering [36,38,48,50] and EM cross-section counts of thick filaments per myofibril [16,20,21]. These data predict an *in vivo* myofibril width of 1.54−1.68 µm, a range that encompasses the measurements obtained by several of the presented methods, including the hardening media embedded isolated-myofibrils that we adopted as our primary standard. We propose that upon sarcolemma removal, isolated myofibrils initially swell due to osmotic overhydration, increasing filament spacing. Embedding in hardening media then imposes compressive forces that reduce this spacing, ultimately yielding diameters (1.54 µm) close to the physiological range, making this method well-suited for accurate morphometric analysis.

In summary, our measurements revealed that factors such as the sex and age of the flies, and the muscle fibre type (DLM, DVM or a mix) have a relatively slight effect on the morphometric parameters, whereas the preparation method might have a more profound effect. Interestingly, sarcomere length is robust against variations in sample preparation, while myofibril diameter can vary in a fairly broad range depending on the dissection method, fixative, fixation duration and the choice of embedding medium. These findings highlight the crucial importance of careful controlling and reporting of our methods to ensure the highest level of consistence in our IFM investigations and to warrant a higher level of comparability between results generated in different laboratories.

### 2.4. Synchronized growth and developmental phases of IFM myofibrillogenesis

During myofibrillogenesis, IFM sarcomeres undergo a substantial, synchronized growth in both length and diameter. Until now, only a few studies have provided detailed insights into this process [1,23,25,37]. While these studies outlined a similar overall pattern of sarcomere growth (electronic supplementary material, figure S3A), some ambiguities remained as to several questions: what is the exact size of the earliest detectable sarcomeres? Is sarcomere growth a continuous process, or does it include pauses? When do sarcomeres reach their final size?

**Figure 3.**
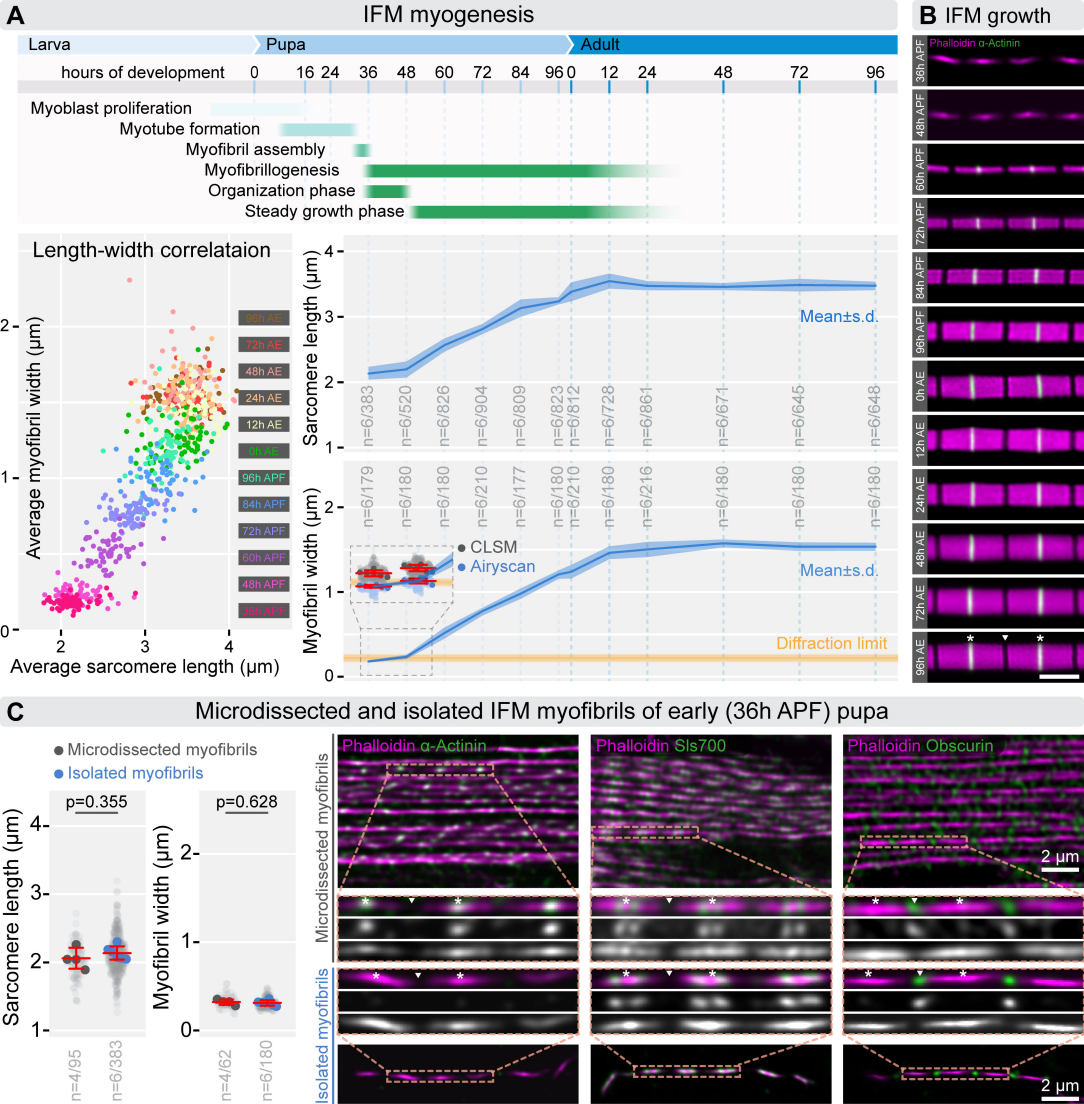
Growth of IFM sarcomeres during myofibrillogenesis. (A) The top panel provides a schematic outlining of the timeline and key steps of IFM myogenesis. In the bottom left, a plot illustrates the correlation between sarcomere length and myofibril width across myofibrillogenesis. Each dot (colour-coded by the time points) represents the mean value of an individual myofibril. The plots on the right display the average sarcomere length and myofibril width measured across 12 time points, from 36 h APF to 96 h AE, following the timeline depicted in the schematic. A yellow line highlights the theoretical resolution limit of optical microscopy, with an inset showing that conventional light microscopy (LSM) overestimates the diameter of early myofibrils. By contrast, Airyscan imaging reveals that these diameters are below the diffraction limit. (B) Images of isolated individual myofibrils showcase the growth of IFM sarcomeres during myofibrillogenesis. F-actin (magenta) is stained with phalloidin, and Z-discs (green) are labelled with α-actinin. (C) Plots demonstrating that sarcomere length and myofibril diameter from young pupae (36 h APF) are consistent whether measured in isolated individual myofibrils or within microdissected muscles. In panel C, light grey dots represent individual measurements, while larger dots show mean values from independent experiments. Error bars indicate the mean and s.d. across experiments, with ‘*n*’ denoting the number of independent experiments/number of individual measurements. The micrographs on the right compare the overall organization of microdissected muscles with isolated individual myofibrils from young pupae (36 h APF). These images also display the localization of sarcomeric markers, including α-actinin, Sls700 and Obscurin. Insets provide higher resolution views to facilitate thorough comparisons. Scale bar: 2 µm. In panels (B) and (C), white asterisks indicate the Z-discs of an exemplary sarcomere, and the position of the M-line is shown by a white triangle. Raw data used to generate the plots presented in this figure are available in the source data file (electronic supplementary material, Fig3SourceData).

**Figure 4.**
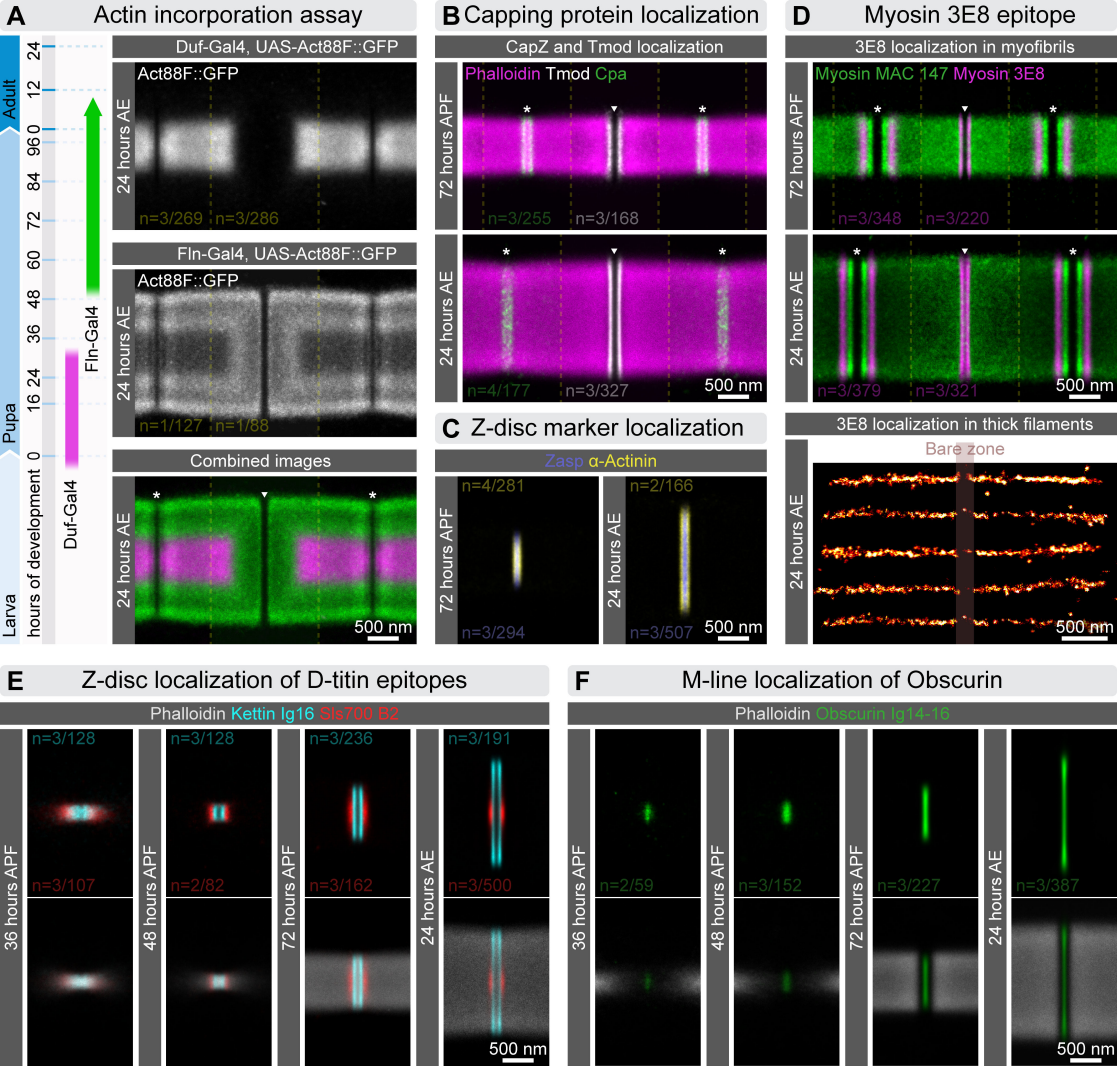
Nanoscopic localization of sarcomeric markers during myofibrillogenesis. (A) The schematic on the left illustrates the restricted temporal activation of the *duf-Gal4* and *fln-Gal4* drivers during myofibrillogenesis. On the right, dSTORM reconstructions display the incorporation of GFP-labelled Act88F monomers into IFM myofibrils using these drivers. (B) Pseudo-multicolour dSTORM reconstructions reveal the localization of Tmod (in white) and Cpa (in green) in IFM myofibrils isolated from pupae (72 h APF) and adult flies (24 h AE). F-actin is labelled with phalloidin (magenta). (C) Pseudo-multicolour dSTORM reconstructions highlight the localization of α-actinin (yellow) and Zasp52 (blue) within the Z-disc of IFM myofibrils from pupae (72 h APF) and adult flies (24 h AE). These proteins were not detected in isolated myofibrils at 36 and 48 h APF, and therefore are not shown in the figure. (D) The top panels present pseudo-multicolour dSTORM reconstructions of IFM myofibrils—myosin epitopes labelled with MAC147 (green) and 3E8 (magenta)—from pupae (72 h APF) and adults (24 h AE). MAC147, a monoclonal antibody against an epitope near the myosin head domain, only yields reliable staining in mature myofibrils (72 h APF and 24 h AE), probably because it recognizes a specific Mhc isoform. For this reason, we omitted images from earlier developmental stages with MAC147. By contrast, the 3E8 antibody provides uniform A-band labelling—excluding the bare zone—in early pupal stages. At 72 h APF and 24 h AE the 3E8 pattern shifts to two narrow stripes beside the bare zone and two broader, diffuse stripes near the A/I junction. This altered staining probably reflects reduced antigen accessibility in the densely packed IFM at later stages, making 3E8 unreliable for thick-filament length measurements then. The bottom panels show dSTORM images of single, isolated thick filaments from adult flies (24 h AE) labelled with 3E8, which binds across the full length of mature thick filaments except at the central bare zone. (E) A series of pseudo-multicolour dSTORM reconstructions reveal the localization of elastic filament epitopes, Kettin Ig16 (cyan) and Sls700 B2 (red), at four different time points during myofibrillogenesis. F-actin is labelled with phalloidin (grey). (F) Series of dSTORM reconstructions illustrate the localization of the central Obscurin Ig14−16 (green) epitope, across four time points of myofibrillogenesis. F-actin is labelled with phalloidin (grey). The white asterisks indicate the Z-discs, while the M-line is shown by a white triangle.

Having our improved measurement methods in hand, we decided to revisit these questions and examine the developmental dynamics of IFM growth. To this end, individual myofibrils were isolated from 12 developmental time points (spanning from 36 h after puparium formation (APF) to 96 h AE) in six independent experiments using mixed-sex wild-type flies (figure 3B). We then measured sarcomere length and myofibril width (figure 3A). Our findings showed that immature, periodic myofibrils appear around 36 h APF at 25°C. We isolated short, intact myofibrils consisting of 4−12 sarcomeres from these young pupae, and found that their length and width were comparable with those measured in microdissected muscles, confirming their structural integrity (figure 3C). Although α-actinin showed a periodic Z-disc pattern in microdissected muscles (as reported before) [51] (figure 3C), this pattern was largely absent in pre-extracted individual myofibrils (figure 3C), suggesting that this protein is loosely associated with the immature Z-discs and can be easily disrupted by detergents. Stable association of α-actinin with Z-discs appeared between 48 and 60 h APF. Among the Z-disc markers we tested, only the Sls700 *Drosophila* Titin-specific B2 and Kettin Ig16 antibodies displayed stable, detergent-resistant Z-disc localization in young pupae (36–48 h APF) (figures 3C and 4E), suggesting that the giant elastic proteins play a key role in early Z-disc and myofibril assembly.

While measuring the diameter of early myofibrils (36 and 48 h APF), we noticed an apparent lower limit which matched the theoretical diffraction limit of conventional fluorescent microscopes (figure 3A). To overcome this limitation, we used Airyscan imaging and found that myofibrils are considerably thinner than previously assessed by regular confocal microscopy [1], though still thicker than measured on EM sections [23] (figure 3A). Between 36 and 48 h APF we only detected a modest increase in sarcomere size, but after this period a marked growth took place without significant changes in the assembly rate up to 96 h APF (figure 3A,B). Subsequently, we observed a sharp increase in sarcomere length between 96 h APF and the time of eclosion (0 h AE), likely due to pre-stretching of IFM myofibrils as the thoracic exoskeleton expands. Our data showed that IFM sarcomere length and diameter generally peak and/or plateau in young adult flies between 12 to 24 h AE (figure 3A). Interestingly, although the causal connection is unclear, we note that it precisely coincides with the isoform switch of Tropomodulin (Tmod), when the approximately 43 kDa isoform responsible for thin filament elongation is replaced by a larger approximately 45 kDa isoform [13].

Based on these growth patterns, we identified two distinct phases of IFM myofibrillogenesis: the organization phase and the steady growth phase (figure 3A). During the organization phase, sarcomere size remained nearly constant, as observed previously [52], while the number of sarcomeres per myofibril increased significantly—from approximately 100 at 36 h APF to about 230 at 48 h APF [1]. After 48 h APF, only a few additional sarcomeres were added, resulting in 260−270 sarcomeres per dorsal longitudinal muscle (DLM) myofibril by 60 h APF [1]. In the steady growth phase, which spans from 48 h APF to 24 h AE, sarcomeres continued to elongate, and myofibril diameter expanded until both reached their mature size in adult flies between 12 to 24 h AE (figure 3A).

### 2.5. Assessing myofilament assembly and elongation during myofibrillogenesis

The growth of sarcomeres in both length and width involves two processes: elongation of the existing myofilaments (thin and thick filaments) and addition of new myofilaments to the sarcomere lattice. Studies, including ours, indicate that thin filaments extend specifically at their pointed ends, while thick filaments grow by adding myosin molecules at their ends. Additionally, newly formed myofilaments integrate at the periphery of the myofibrils, gradually expanding the structure in radial direction (figure 4A) [13,16].

**Figure 5.**
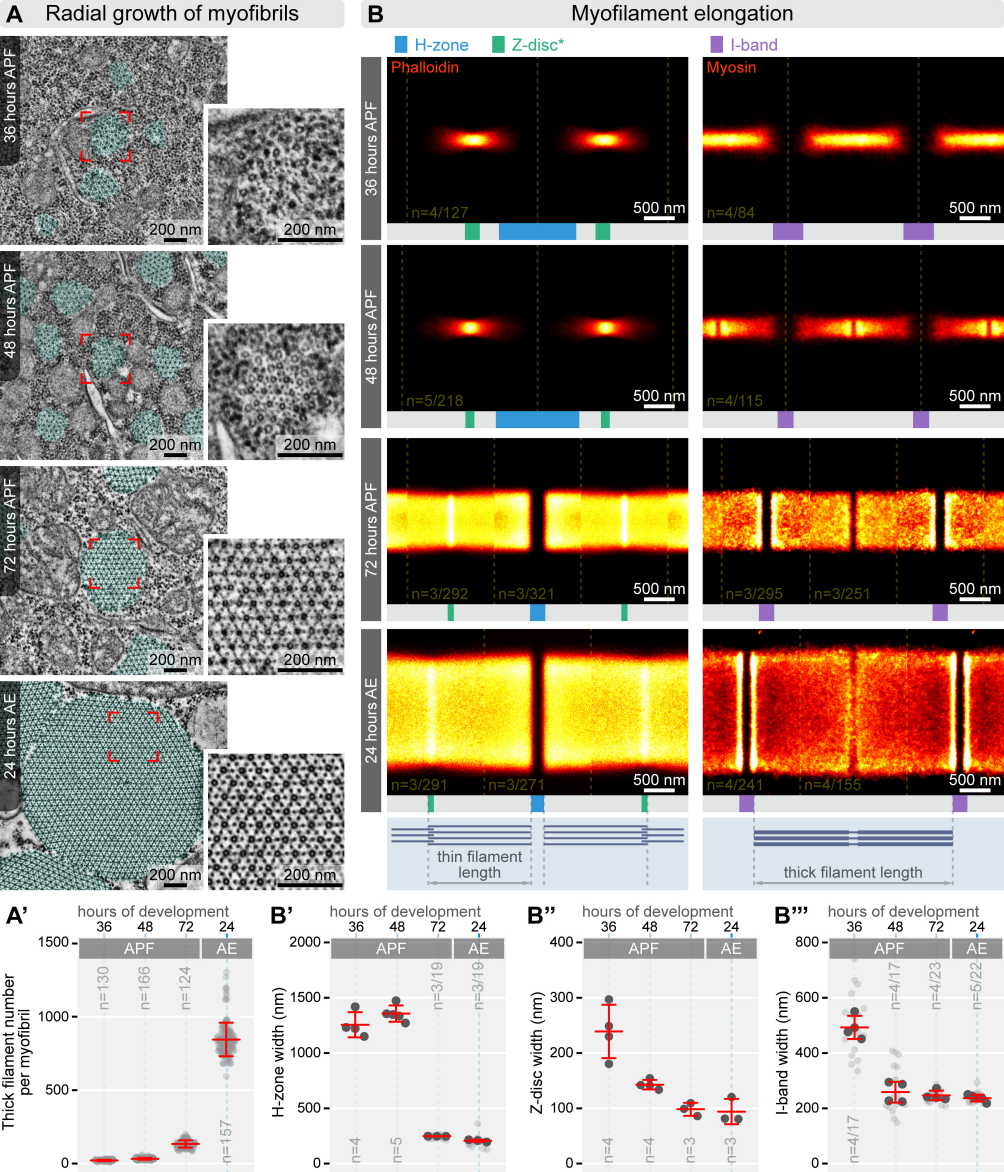
Characterization of myofilament number and length during myofibrillogenesis. (A) TEM cross-sections of DLM fibres highlight the radial growth of myofibrils (highlighted in cyan) throughout myofibrillogenesis. Higher resolution insets on the right showcase changes in the lattice organization of myofilaments. (B) The accompanying plot displays the average number of thick filaments observed in the TEM cross-sections of myofibrils, with the mean and s.d. indicated. (C) Averaged dSTORM reconstructions illustrate the growth and spatial organization of thin filaments (stained with phalloidin, on the left) and thick filaments (labelled with myosin antibodies, on the right) during myofibrillogenesis. (D–F) Plots showing measurements of H-zone width (D), Z-disc width (E) and I-band width (F) derived from dSTORM images. In panels (B), (D), (E) and (F) light grey dots represent the mean values for individual myofibrils, while dark grey dots indicate the mean values from independent experiments. The mean and s.d. of these experiments are provided. ‘*n*’ refers to the number of independent experiments/number of individual measurements, when relevant. Raw data used to generate the plots presented in this figure are available in the source data file (electronic supplementary material, Fig4SourceData).

**Figure 6.**
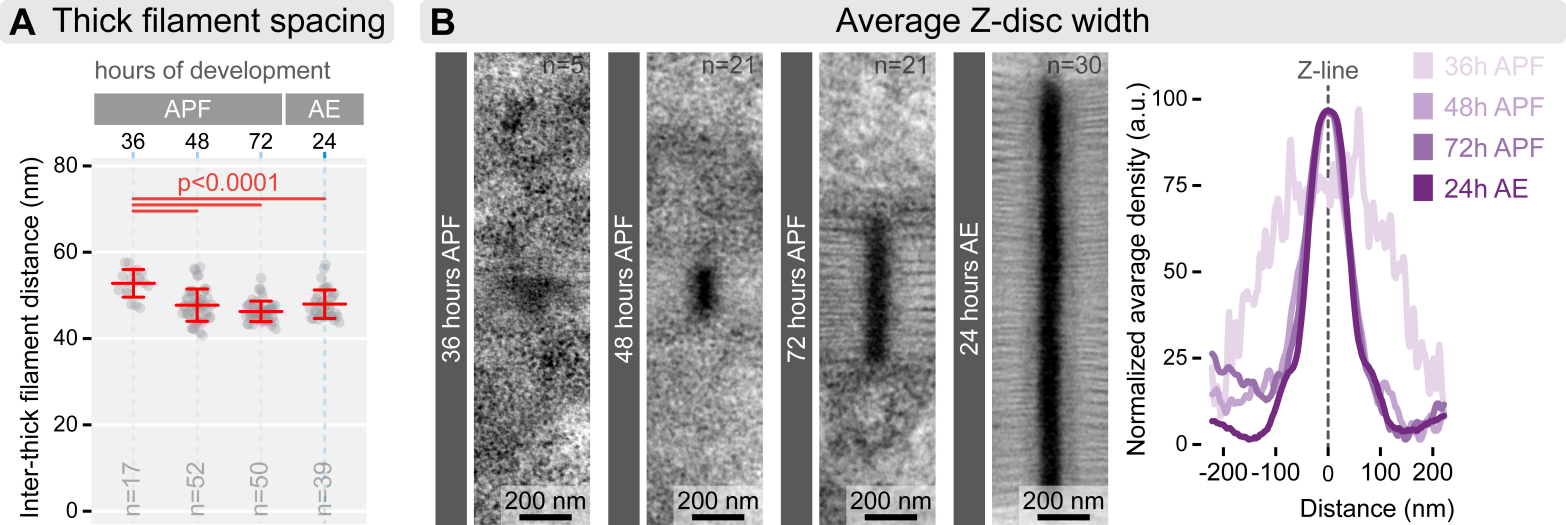
Myofilament lattice spacing and Z-disc width during myofibrillogenesis. (A) The plot shows the centre-to-centre spacing between thick filaments at four time points during myofibrillogenesis. At 36 h APF, the spacing is around 53 nm, which significantly decreases to approximately 46−48 nm by 48 h APF, and this spacing remains constant for the rest of development. Statistical analysis was performed using one-way ANOVA with Tukey’s multiple comparison test. Error bars represent the mean and s.d., and ‘*n*’ refers to the number of myofibrils analysed. (B) Averaged TEM images on the left illustrate the Z-body/Z-disc morphology at four designated time points during myofibrillogenesis. The plot on the right shows changes in the Z-body/Z-disc density profile throughout the process. At 36 h APF, only diffuse Z-bodies are visible, which then organize into compact Z-discs by 48 h APF. While the height of the Z-disc increases significantly, Z-disc width decreases only slightly from this point onward. Raw data used to generate the plots presented in this figure are available in the source data file (electronic supplementary material, Fig6SourceData).

While standard fluorescent microscopy is sufficient to measure sarcomere length and diameter, determining the precise length and number of the myofilaments requires higher resolution imaging. For this, we utilized fluorescent nanoscopy to measure myofilament length and electron microscopy to count myofilaments across four developmental stages (from 36 h APF to 24 h AE). To examine myofilament number in myofibrils and arrangement during myofibrillogenesis, we analysed cross-sections of DLM myofibrils using transmission electron microscopy (figure 5A,B). At 36 h APF, myofibrils appeared as disorganized clusters of filaments, with an average of 23 thick filaments per myofibril. By 48 h APF, myofibrils began to adopt a more structured arrangement, establishing a lattice spacing typical of mature myofibrils (figure 6A). The number of thick filaments increased to an average of 32, forming a nearly perfect hexagonal pattern with a 1:3 ratio of thick to thin filaments, even though irregularities were still observed at the edges. At 72 h APF, the lattice structure became fully organized, resembling the adult form, with an average of 134 thick filaments. In adult flies (24 h AE), the number of thick filaments per sarcomere averaged around 846, consistent with previous research [16,20,21].

Exploiting that thin filaments are organized such that their pointed ends face the edge of the H-zone, while their barbed ends overlap at the Z-disc, their length can be measured by visualizing capping proteins like CapZ (a barbed end capper) and Tmod (a pointed end capper). This approach is effective from the later pupal stages (72 h APF) till the young adults (24 h AE; [7]), where the Tmod and CapZ (i.e. Cpa) signals form well-defined lines (figure 4B). However, in the IFM of early pupae (36 and 48 h APF), both signals are weak and disordered, making such measurements impractical. Instead, we used phalloidin-labelled myofibrils and visualized them with dSTORM microscopy to measure the length of the thin filament arrays (figure 5C). Phalloidin specifically binds to F-actin and, due to its small size, provides relatively uniform labelling across the entire myofibril without introducing 'linkage errors'. Thin filament length could be deduced from sarcomere length, the width of the H-zone and of the Z-disc overlap (figure 5C*–*E; electronic supplementary material, figure S4A).

At 36 h APF, thin filaments had not yet aligned in perfect register at the Z-disc, with most measuring less than 560 nm in length—and exhibiting considerable variability (figure 5; electronic supplementary material, figure S5). The overlap of the thin filaments at the Z-disc was approximately 240 nm, consistent with the width of the electron-dense Z-bodies observed in longitudinal EM sections of myofibrils at this stage (figure 6B). While D-Titin (labelled with Sls700 B2 and Kettin Ig16) was already arranged in a bipolar pattern, the distribution appeared rather diffused (figure 4E). Additionally, the Z-discs lacked stable binding of α-actinin and Zasp52 (figure 4C). By 48 h APF we measured a thin filament array length of approximately 490 nm (electronic supplementary material, figure S5), which is somewhat shorter than at 36 h APF. Nevertheless, we think it very unlikely that the actin filaments indeed shrink, instead we suspect that by 48 h APF the sarcomeres attain a higher structural regularity than at 36 h APF, which makes the apparent length of the thin filament array shorter without significant changes in filament length. Consistent with this idea, by 48 h APF the Z-discs became more structured, with a reduced thin filament overlap of approximately 140 nm, resembling the mature configuration, as also seen in longitudinal EM sections (figure 6B). The D-Titin markers also appeared more compact, likely indicating that filaments were aligning in register (figure 4E). However, stable associations of α-actinin and Zasp52 were still absent. At 72 h APF the thin filament array adopted a structure closely resembling its mature form, now with stable α-actinin and Zasp52 associations (figure 4C). Thin filaments extended to approximately 1330 nm (electronic supplementary material, figure S5), and the overlap at the Z-disc was reduced to approximately 98 nm, which is only slightly larger than the approximately 94 nm observed in mature sarcomeres. Longitudinal EM sections further supported these findings (figure 6B), and the Z-disc overlap size was consistent with measurements from the IFM of other species obtained through cryo-EM reconstructions [53,54]. Finally, by 24 h AE, thin filaments reached their final length of approximately 1680 nm (electronic supplementary material, figure S5), and all Z-disc markers exhibited their fully matured, double-line pattern (figure 4C,E) [7].

To track the process of thick filament elongation we used two different myosin antibodies at different developmental stages: Myosin 3E8 for 36 h and 48 h APF, and MAC147 for 72 h APF and 24 h AE (figure 5C). Myosin 3E8 labels the A-band uniformly in early stages but shows altered staining in later stages due to reduced antigen accessibility (figure 4D). By contrast, MAC147 [55] reliably stains mature myofibrils, but it is less suitable for staining in earlier stages likely due to isoform specificity. The length of the thick filaments was calculated based on the average sarcomere length and the I-band width measured on the nanoscopic reconstructions (figure 5C,F; electronic supplementary material, figure S4A). At 36 h APF, the thick filament array appeared fairly well-defined but it seemed somewhat misaligned since no bare zone was visible at the M-line (figure 5C). The array measured about 1640 nm in length on average (electronic supplementary material, figure S5), although individual thick filaments were probably slightly shorter. At this stage, Obscurin was only weakly detected using an antibody against its central domains (Ig14−16) (figure 4F). By 48 h APF, the average thick filament length increased to approximately 1940 nm (electronic supplementary material, figure S5), and a distinct bare zone became visible at the M-line, suggesting that the filaments were aligning in register. The I-band width at this point already resembled that of mature sarcomeres (figure 5C,F). Additionally, the Obscurin Ig14−16 signal was now clearly seen as a single band along the M-line, as in previous reports for the adult muscles [7] (figure 4F). At 72 h APF, both the width of the thick filament array and the length of the individual filaments had grown significantly, the latter reaching approximately 2570 nm (figure 5C,F; electronic supplementary material, figure S5). Finally, by 24 h AE, the thick filaments attained their final length of approximately 3240 nm, consistent with earlier studies [18,19]. At this stage, the Obscurin pattern became more compact along the longitudinal axis of the sarcomeres, corresponding to a narrowed H-zone (figures 5C,D and 4F).

Thus, the application of two high resolution microscopy methods (TEM and dSTORM) allowed us to collect filament level information about the precise number and length of the myofilaments in the developing IFM. These data, combined with entire sarcomere level measurements, provided us with all knowledge to accurately determine the dimensions of every sarcomeric region, and served as an excellent starting ground for reconstruction of the IFM myofilament lattice.

### 2.6. Myofilament lattice model of developing IFM sarcomeres

**Figure 7.**
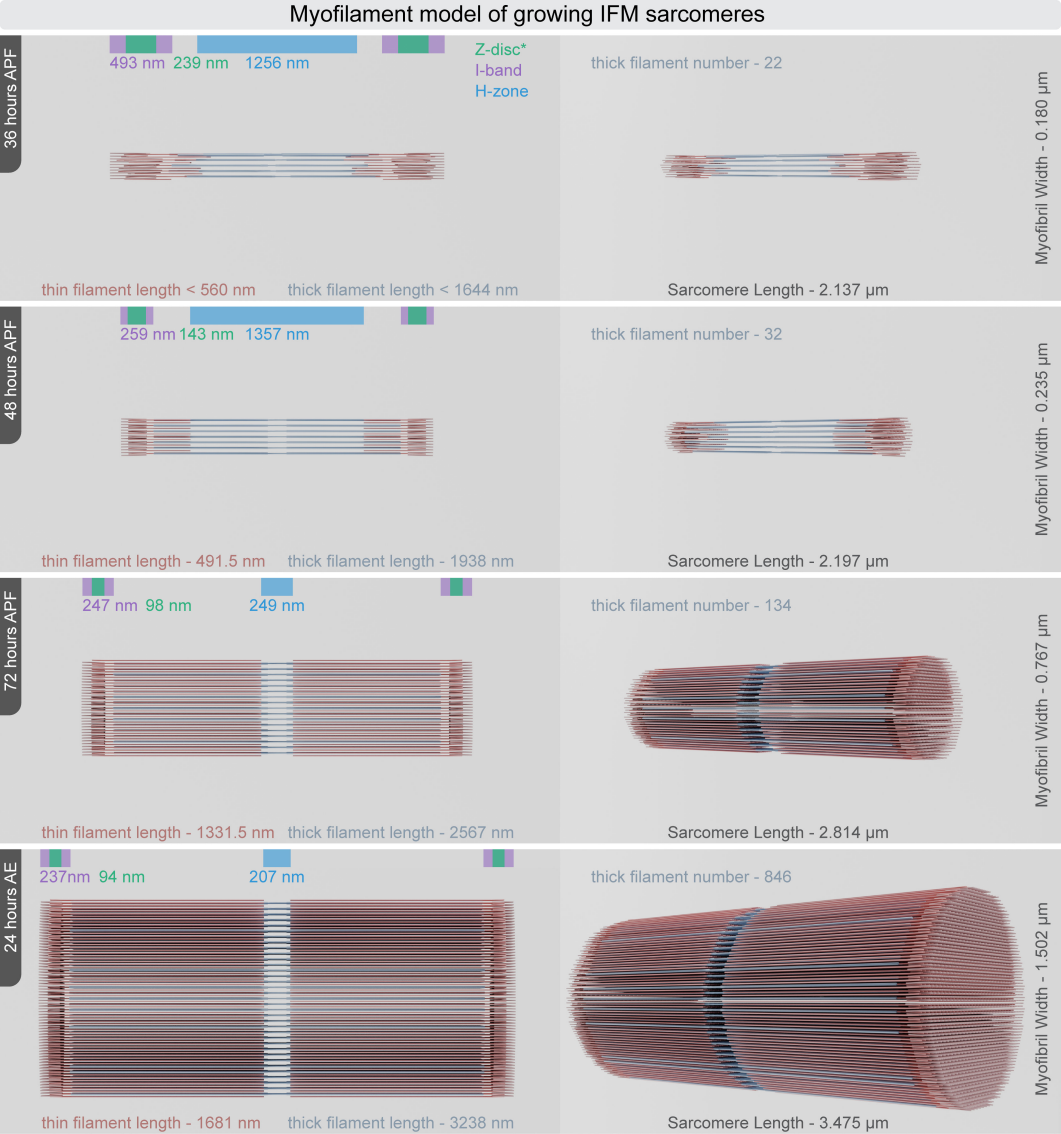
Myofilament model of developing IFM sarcomeres Three-dimensional scaled models of sarcomeres illustrate the average number, size and arrangement of thick filaments (blue) and thin filaments (red) during myofibrillogenesis. The models on the left present orthographic views, while those on the right display rotated perspective views of the sarcomeres. Data presented in this figure are available in a table form in the source data file (figure 7sourcedata).

Using morphometric measurements from confocal, TEM and dSTORM microscopies, we generated sarcomere blueprints that represent the average sarcomere structure at specific developmental stages (see an example in electronic supplementary material, figure S4A). In the *Drosophila* IFM, myofilaments form a regular hexagonal lattice, maintaining a 3 : 1 ratio of thick to thin filaments. These filaments are arranged in a MyAc layer, with thin filaments consistently positioned at the midpoint between two thick filaments [56]. Incorporating these structural constraints, we developed simplified models of average sarcomeres in cross-sectional and longitudinal views (see an example in electronic supplementary material, figure S4B). From these two-dimensional representations, we constructed scaled three-dimensional models (figure 7; electronic supplementary material, video S1–S4), assuming a symmetry for thin filaments in the Z-discs, similar to what is observed in the IFM of other insect species [53,54,57]. These models represent our current understanding of how the myofilament arrays in IFM sarcomeres expand in length and diameter during myofibrillogenesis.

In addition to providing direct measurements of myofilament length, number and spatial arrangement, our sarcomere-level models also allowed us to track how these parameters change over time and to calculate myofilament elongation or assembly rates. Accordingly, during the first phase, from 36 to 48 h APF, incorporation of a single thick filament (or six thin filaments) requires approximately 65 min. Between 48 and 72 h APF, this process accelerates reducing the integration time to around 15 min, and from 72 h APF to 12 h AE, the incorporation time further decreases to about 4.5 min, eventually slowing down when the sarcomeres attain their mature size. These data can now be used as a reference point for comparisons of the developmental dynamics of IFM growth in different conditions.

## 3. Summary

The IFM is a broadly used model for investigating the molecular mechanisms underlying sarcomere structure and muscle development due to its highly regular, uniform and easily observable architecture. While this structural uniformity is not in question, we noted that reported measurements often exhibit significant variability as to the basic sarcomeric parameters. In this study, we identified several factors influencing these measurements and addressed them systematically. We found that a part of the differences can be attributed to the inaccuracy of the measurement methods. In addition, we revealed that although sarcomere length is consistent across sexes, muscle fibre types and experimental conditions, myofibril width is highly sensitive to the experimental methods, and it even exhibits a slight difference between DLM and DVM fibres.

To achieve precise and high-throughput measurements, we developed a software tool that segments myofibrils from micrographs, applies spline fitting and fits the appropriate models to intensity profiles to accurately calculate sarcomere length and myofibril width. The accuracy of this new tool was validated using simulated IFM images with known dimensions, while its precision was confirmed by comparisons with manual measurements on blinded datasets.

Transmission electron microscopy was applied to directly measure the number of myofilaments in IFM myofibril cross-sections during myofibrillogenesis. Additionally, dSTORM microscopy was used to determine the length of thin and thick filaments by measuring the width of the H-zone, I-band and Z-disc. These measurements were integrated into comprehensive models describing sarcomere growth at the level of individual myofilaments. This model serves two primary purposes:

1. *Providing a spatial reference framework*. While fluorescent super-resolution microscopy precisely localizes sarcomeric molecules, interpreting these data can be challenging without a broader context of sarcomere ultrastructure. Our models offer a spatial framework that leverages the uniformity, regularity and symmetry axes of the sarcomere to help to accurately position specific molecules within the myofilament array [7].
2. *Quantifying sarcomere growth dynamics*. Beyond providing critical spatial information, our data allowed us to determine the dynamics of filament incorporation. This knowledge can now be used as a reference for studying the temporal aspects of this highly controlled developmental process.

Overall, we believe that this model could provide a foundation for future studies exploring the molecular mechanisms of myofilament elongation and formation. Moreover, it holds a great potential for a wide range of applications in understanding sarcomere dynamics at the molecular level.

## 4. Study limitations

Our study is focused on describing the growth of IFM sarcomeres during myofibrillogenesis at the level of individual myofilaments. Additionally, we developed a user-friendly software tool for precise sarcomere size measurements and demonstrate that these measurements are sensitive to varying conditions. Whereas, this tool can be used successfully on whole muscle fibre preparations as well, our pipeline was intentionally optimized for individual IFM myofibrils ensuring higher measurement precision in our hands than other type of preparations. Thus, we predict that future work will be required to extend it to sarcomeres from other muscle tissues or species. Nevertheless, our study exemplifies a workflow how to measure sarcomere dimensions precisely. With some variations, it should be possible to adopt it for other muscles, including vertebrate and human striated muscles. To facilitate this and to enhance the accessibility and usability of this dataset, we welcome any feedback and suggestions from researchers in the field.

## 5. Material and methods

### 5.1. *Drosophila* stocks

Flies were raised on cornmeal medium and maintained at 25°C under standard laboratory conditions. Morphometric analysis of sarcomere growth was conducted using the *w^1118^* strain. Stocks of *Oregon-R*, *Canton-S* and *Mef2-GAL4* (BDSC no. 27390) [58], were used as controls. For staging, white pre-pupae or freshly hatched flies were collected and maintained until they reached the desired age. In the actin incorporation assay, the *UAS-Act88F::GFP* transgene (BDSC no. 9253) [59] was expressed using either the *duf-Gal4* [60] or *fln-Gal4* [61] driver.

### 5.2. Sample preparation for fluorescent microscopy

#### 5.2.1. IFM agarose sectioning

Thoraces were separated from the heads and abdomens using fine forceps, with the heads and abdomens discarded. The isolated thoraces were immediately placed in 4% paraformaldehyde (Thermo Fisher Scientific; paraformaldehyde, 16%) in relaxing solution (100 mM NaCl, 20 mM NaPi at pH 7.0, 5 mM MgCl_2_, 5 mM EGTA and 5 mM ATP) and fixed overnight at 4°C. After a brief rinse in PBS, the thoraces were submerged in warm 5% agarose (Lonza, SeaKem LE Agarose) prepared in PBS and positioned appropriately. Once the agarose solidified, the blocks were glued to a sample holder and inserted into a vibratome (Microm HM 650 V). The lateral side of the embedded thorax was oriented towards the vibratome blade, which was submerged in PBS. Using the vibratome, 120 µm thick sections were collected and stored in PBS at 4°C. Standard immunohistochemistry procedures were then applied to the sections.

#### 5.2.2. IFM microdissection

Thoraces were separated from the heads and abdomens using fine forceps, discarding the heads and abdomens. The thoraces were then bisected along the sagittal midline and immediately fixed in 4% paraformaldehyde (Thermo Fisher Scientific; paraformaldehyde, 16%) prepared in relaxing solution for 20 min on ice. After fixation, IFM muscle fibres were carefully detached from the hemithoraces, transferred to PBS, and stored at 4°C. Standard immunohistochemistry procedures were subsequently applied to the isolated fibres.

#### 5.2.3. IFM individual myofibril preparation

Individual myofibrils were isolated from the IFM using a modified protocol based on [62]. Briefly, bisected hemithoraces were incubated on ice in relaxing solution containing 50% glycerol for 2 h. The DLM and/or DVM fibres were then carefully dissected from the hemithoraces and dissociated in an Eppendorf tube by pipetting with 0.5% Triton X-100. The dissociated myofibrils were centrifuged at 10 000 × *g* for 2 min at 4°C, and the pellet was re-suspended in 200 µl of 1× relaxing solution by pipetting. This centrifugation and washing process was repeated twice. After the final centrifugation, the fibres were re-suspended in relaxing solution, and 20 µl of the suspension was placed on a glass coverslip and fixed for 20 min (alternatively for 10 or 60 min) either with 4% paraformaldehyde (Thermo Fisher Scientific; paraformaldehyde, 16%) or with 2% glutaraldehyde (Electron Microscopy Sciences; glutaraldehyde, 8%) in relaxing solution (see [63] for more details).

#### 5.2.4. Thick filament isolation

Thick filaments were isolated from the IFM following the protocol outlined in [18]. In summary, individual myofibrils (prepared as described above) were subjected to mild Calpain digestion to separate the thick filaments from the Z-disc [64,65]. The myofibrils were resuspended in 30 µl of a solution containing 20 mM imidazole (pH 6.8), 1 mM EDTA, 1 mM EGTA, 5 mM β-mercaptoethanol and 30% glycerol, along with 15 mM Calpain-1 (Merck). CaCl_2_ was added to achieve a final concentration of 2 mM, and the mixture was incubated at room temperature for 30 min. To halt the digestion, 100 µl of relaxing solution and 100 mM Calpain Inhibitor I (Merck) were added. The suspension was then passed through a 20G needle five times to separate the filaments and centrifuged at 1500 × *g* for 3 min to remove undigested myofibrils. The resulting suspension was applied to a glass coverslip and fixed for 20 min with 4% paraformaldehyde (Thermo Fisher Scientific; paraformaldehyde, 16%). Isolated and fixed thick filament samples were then subjected to standard immunohistochemistry procedures.

### 5.3. Immunohistochemistry

Fixed tissues were washed three times and blocked in PBS-BT for 2 h at room temperature (30 min for individual myofibril samples). The following primary antibodies were applied in blocking solution and incubated overnight at 4°C:

α-actinin (DSHB 2G3-3D7, mouse, 1 : 100)
Cpa (gift from Florence Janody, rabbit, 1 : 100 [66])
GFP (Abcam ab13970, chicken, 1 : 1000)
Kettin Ig16 (DSHB BB17/29.5, rat, 1 : 200)
Myosin 3E8 (DSHB 3E8-3D3, mouse, 1 : 1000)
Myosin MAC147 (DSHB BB7/21.14, rat, 1 : 1000)
Obscurin Ig14-16 (gift from Belinda Bullard, rabbit, 1 : 400 [62])
Sls700 B2 (gift from Belinda Bullard, rabbit, 1 : 200 [67])

After additional washes, secondary antibodies were applied for 2 h at room temperature. Detection was carried out with highly cross-absorbed goat anti-rabbit, anti-mouse, anti-rat or anti-chicken IgG secondary antibodies conjugated to Alexa Fluor 405, Alexa Fluor 488, Alexa Fluor 546 or Alexa Fluor 647 (Life Technologies, 1 : 600). F-actin was labelled with Alexa Fluor 488-, Alexa Fluor 546- or Alexa Fluor 647-phalloidin (Life Technologies, diluted in methanol, 1 : 200 or 1 : 800 for dSTORM imaging). After thorough washes, the samples were mounted in either ProLong Gold (P36930; Life Technologies), Vectashield (H-1000; Vector Laboratories), glycerol (90% glycerol, 10% PBS), or stored in PBS until dSTORM imaging.

### 5.4. Confocal laser scanning microscopy

Confocal images were acquired using a Zeiss LSM800 Airyscan microscope with either a Plan-Apochromat 63×/1.40 Oil DIC M27 or an EC-Plan-Neofluar 10×/0.30 M27 objective lens. Images were captured at the Nyquist rate to ensure optimal resolution, utilizing the full dynamic range of the GaAsP or Airyscan detectors.

### 5.5. Super-resolution dSTORM imaging

Super-resolution imaging was conducted as previously described [7,63]. Imaging was performed using a custom-built inverted microscope system based on a Nikon Eclipse Ti-E frame, equipped with a Nikon CFI Apo 100×, NA = 1.49 objective lens. Excitation was provided by a 647 nm laser (MPB Communications, Pmax = 300 mW), with the intensity adjusted to 2−4 kW cm^−2^ at the sample plane via an acousto-optic tunable filter (AOTF). A 405 nm laser (Nichia, Pmax = 60 mW) was used for molecule reactivation. Images were captured with an Andor iXon3 897 BV EMCCD camera (512 × 512 pixels, 16 μm pixel size) as frame stacks for dSTORM imaging at reduced image size. A fluorescence filter set (Semrock, LF405/488/561/635 A-000) and an additional emission filter (AHF, 690/70 H Bandpass) were used to separate excitation and emission light. During imaging, the perfect focus system of the microscope maintained sample focus with precision < 30 nm. Before imaging the storage buffer was replaced with a GLOX switching buffer [68], and the sample was mounted onto a microscope slide. Typically, 20 000−50 000 frames were captured with an exposure time of 20−30 ms. Image stacks were analysed using rainSTORM localization software [69], where single-molecule images were fitted with a Gaussian point spread function, and the centre positions of fluorescent molecules were determined. Localization data were filtered based on intensity, precision (<20 nm), and standard deviation (0.8 ≤ σ ≤ 1.0). Drift caused by mechanical or thermal effects was corrected using a correlation-based blind drift correction algorithm. See [63] for more details.

### 5.6. Transmission electron microscopy

Dissected hemithoraces were fixed overnight at 4°C in a solution containing 3.2% paraformaldehyde, 0.5% glutaraldehyde, 1% sucrose and 0.028% CaCl₂ in 0.1 N sodium cacodylate buffer (pH 7.4). The samples were then washed twice in 0.1 N sodium cacodylate buffer (pH 7.4) overnight at 4°C, washed for 15 min in distilled water, and post-fixed for 1 h in 1% osmium tetroxide (Sigma-Aldrich) in distilled water. After osmium fixation, the samples were rinsed in distilled water for 10 min and dehydrated in a graded ethanol series (50% to 100%) for 10 min at each concentration (twice in 100%). Following dehydration, the muscles were treated with propylene oxide for 5 min (Molar Chemicals) and embedded in an epoxy-based resin (Durcupan ACM; Sigma-Aldrich). Mixture of propylene oxide and epoxy-based resin (3 : 1, 1 : 1, 1 : 3) were added to the samples for 1−1−1 h. After, samples were processed through pure resin twice for 1 h, and through pure resin again in room temperature overnight. The resin blocks were polymerized at 56°C for 48 h. Once polymerized, the blocks were etched, and 50 nm ultrathin sections were cut using a Leica Ultracut UCT ultramicrotome. The sections were mounted on single-hole, formvar-coated copper grids (Electron Microscopy Sciences) and stained to enhance contrast. Staining was performed using 2% uranyl acetate in 50% ethanol and 2% lead citrate in distilled water (both from Electron Microscopy Sciences). The ultrathin sections were examined using a JEM-1400Flash transmission electron microscope (JEOL). Muscle cross-sections were systematically screened at low magnifications (500−2000×), and images were captured at higher magnifications—12 000× for cross-sections and 8000× for longitudinal sections. Images were recorded as 16-bit greyscale files using a 2k × 2k high-sensitivity scientific complementary metal-oxide-semiconductor (sCMOS) camera (Matataki Flash, JEOL) and saved in tagged image file format.

### 5.7. Image processing and analysis

#### 5.7.1. Confocal images

Sarcomere length and diameter were measured directly from raw .czi files using a custom algorithm called *Individual Myofibril Analyser* (IMA). Isolated myofibrils were analysed in Automatic Mode, while intensity profiles were manually selected for microdissected or sectioned muscle samples. The source code and a detailed user guide are available online (https://github.com/GorogPeter94/Individual-Myofibril-Analyser-IMA-/tree/main). The presented images were restored using Huygens Professional Software 23.10 (Scientific Volume Imaging). Optical sections were displayed as average projections, with brightness and contrast linearly adjusted in Fiji [70].

#### 5.7.2. TEM images

For presentation purposes, the contrast of TEM images was enhanced using the Enhance Local Contrast (CLAHE) method in Fiji [70]. Myofilament numbers were manually quantified in raw images using Fiji’s Multi-point tool. The centre-to-centre distance between thick filaments was measured following the methodology detailed by [21]. Average Z-disc images were created using TEM images of longitudinal DLM sections. Regions containing Z-bodies or Z-discs were then selected and compiled into a single stack. The stacks were aligned laterally with subpixel registration using the ‘Align slices in stack’ function of the Template Matching plugin [71] in Fiji, and average projections were subsequently generated.

#### 5.7.3. dSTORM images

The filtered and drift-corrected single-molecule localization data were processed as detailed in [7,63,72]. Briefly, visualization and quantitative analysis were performed using IFM Analyser v2.1. To create averaged structures, the software’s merge function was used to align localizations along the symmetry axes of the H-zone/I-band before averaging. A super-resolved image with a 10 nm pixel size was then generated from the merged event lists. Relying on the symmetry axes of the H-zone and I-band, we aligned independently averaged protein densities to generate pseudo-multicolour representations. Quantitative measurements were conducted on averaged structures derived from either a single myofibril or, in cases of high noise, from multiple myofibrils captured within a single experiment.

### 5.8. Simulated IFM images

Ground truth images of phalloidin and α-actinin labelled myofibrils were generated as 2D projections of uniformly labelled cylindrical objects, with overlapping regions representing Z-discs and gaps representing H-zones. These images were scaled and the image formation process was simulated in Fiji [70]. Uneven labelling was modelled by applying a Gaussian noise (sigma = 2). The point spread function (PSF) of the microscope was simulated by convolving the images with a 2D Bessel function (numerical aperture: 1.4, wavelength: 525 nm) using Fiji’s MosaicSuite. Shot noise was introduced by adding Poisson noise.

### 5.9. Thick and thin filament length calculations

The following equations were used to calculate the length of thick and thin filaments

Thick filament length ($L_{thick}$):


$$L_{thick}\;=\;L_{sarcomere}-\;\frac{\;2\;*\;W_{I-band}\;}{2}$$

where

$L_{sarcomere}$ is the average sarcomere length

$W_{I-band}$ is the average width of the I-band.

Thin filament length ($L_{thin}$):


$$L_{thin}\;=\;\frac{L_{sarcomere}}{2}\;-\;\frac{W_{H-zone}}{2}\;+\;\frac{W_{Z-disc}}{2}$$

where

$L_{sarcomere}$ is the average sarcomere length

$W_{H-zone}$ is the average width of the H-zone

$W_{Z-disc}$ is the average width of the Z-disc.

### 5.10. Data analysis and figures

Data collection and organization were carried out in Microsoft Excel, while statistical analysis and graph creation were performed using GraphPad Prism 8 or R. When presenting multiple independent experiments, we used superplots where individual measurements are shown as pooled data points to illustrate spread, distribution and sample size, while the mean from each experiment is overlaid with error bars to indicate inter‐experiment variability. We report sample sizes for both individual measurements and independent experiments. Experiments were considered independent when specimens came from different parental crosses, and each experiment included approximately six animals to capture individual variability. The raw data used to generate all plots presented in the figure panels are available in the corresponding source data files. Data normality was assessed using the D’Agostino and Pearson or the Shapiro–Wilk test. The specific statistical tests applied are detailed in the figure legends. A *p*-value of <0.05 was considered statistically significant. Figures were created and finalized in Illustrator (Adobe), while 3D models and animations were produced using Blender 4.0 (Blender Foundation).

## Data Availability

All measured values are included in the source data files. Supplementary material is available online [73].
